# Pathology and Prevalence of Antibiotic-Resistant Bacteria: A Study of 398 Pet Reptiles

**DOI:** 10.3390/ani12101279

**Published:** 2022-05-17

**Authors:** Romeo T. Cristina, Rudolf Kocsis, János Dégi, Florin Muselin, Eugenia Dumitrescu, Emil Tirziu, Viorel Herman, Aurel P. Darău, Ion Oprescu

**Affiliations:** 1 Faculty of Veterinary Medicine, Banat University of Agricultural Science and Veterinary Medicine “King Michael I of Romania”, Calea Aradului 119, 300645 Timișoara, Romania; kocsis_rudolf@yahoo.com (R.K.); janosdegi@usab-tm.ro (J.D.); florinmuselin@usab-tm.ro (F.M.); eugeniadumitrescu@usab-tm.ro (E.D.); emiltiziu@usab-tm.ro (E.T.); viorel.herman@fmvt.ro (V.H.); ioan.oprescu@fmvt.ro (I.O.); 2 Tierarztpraxis Kocsis, Gustav-Vogelmann Straße 5, 71543 Wüstenrot-Finsterrot, Germany; 3 Faculty of Biology, Western University “Vasile Goldis”, Revolutiei Blvd. no. 94, 310025 Arad, Romania; petru.darau@uvvg.ro

**Keywords:** antibiotic sensitivity, bacterial load, pathology, pet-reptiles

## Abstract

**Simple Summary:**

The importance of the present study stems from the fact that some isolated bacterial pathogens from pet reptiles represent a high zoonotic risk, with the human owners of these pets also being considered potential reservoirs for resistant bacteria. This research emerged for practical reasons and the need for more information on this subject. The observations were focused on parallel analysis of the pathologies responsible for major diseases in reptile species kept in terrariums as pets. The aim of this study was to obtain a deeper understanding of the main features of antibiotic therapy and antibiotic resistance in these species. In reptilian species, the simplest and easiest way to combat resistance is by the completion of an antibiogram, it is a current and reliable method to prepare the best anti-infective remedy and to preserve the antimicrobial agents available for therapy. However, generally, the antibiotic dosages used in reptiles are either extrapolated from human medicine or empirically assumed according to the reptile species. A reason for this could be the lack of a proper anti-infective agent (or group) for the particular pathologies affecting reptiles, which may often cause the inaccurate use of another related class of antimicrobials for long periods.

**Abstract:**

Reptiles are potential reservoirs of bacteria that could be transmitted, thus becoming a zoonotic hazard. (1) Background: This three-year investigation surveyed the pathological status of 398 pet reptiles: chelonians, snakes (venomous/non-venomous), and lizards. The main pathological entities found were related to the skin, the sensory organs, the digestive system, the respiratory system, the cardiovascular system, the urinary system, the genitalia, the osteo–muscular tract, surgical issues, tumors, and intoxications. (2) Methods: In 25 individuals treated with antibiotics, no clinical healing was recorded, for this reason, an antimicrobial resistance profile analysis of the 43 samples gathered was processed. An antibiogram was performed using the VITEK^®^2 ID-GP (bio-Mérieux, Marcy l’Etoile, France) automated platform, with 22 bacterial strains being isolated. (3) Results: The statistics (ANOVA) revealed that the most common disease category was diseases of the digestive system, followed by diseases of the skin, respiratory system, nervous system, and reproductive system. A significant correlation (*p* < 0.01) between disease incidence and reptile species was reported, with correlations found between all species and diseases diagnosed. The most common bacteria isolated were *Enterococcus faecalis*, Pseudomonas aeruginosa, Stenotrophomas (Xanthomonas) maltophilia, Escherichia coli, Klebsiella oxytoca, and *Salmonella* spp., but *Beta-hemolytic Streptococcus*, Staphylococcus aureus, Citrobacter spp., and Proteus spp. were also identified. (4) Conclusions: These microorganisms revealed degrees of resistance against penicillins, cephalosporins, macrolides, lincosamides, aminoglycosides, and tetracyclines. The animals can be categorized according to their sensitivity to diseases in the following order (most sensitive to least sensitive): chelonians, venomous snakes, non-venomous snakes, and lizards.

## 1. Introduction

The pathology of reptiles is currently a field of interest. These interesting animals and their needs, specific pathologies, and particularities have been studied during the last decade by specialists in the field, and information on these topics is valued highly [1]. In many cases, the owners remain uninformed about the risks associated with handling reptiles. A potential result of continued contact with reptiles is the higher probability for the transfer of diseases, particularly those of bacterial origin [2,3,4], as well as mycotic [5], viral [6,7], and parasitic and related diseases [8,9].

Infectious diseases, frequently triggered by opportunistic agents, often infect immune-suppressed hosts, and are the main reasons for illness and mortality [10,11]; thus, antimicrobial therapy is an essential element in the medical management of reptiles affected by bacterial or mycotic infections [12,13]. Nevertheless, choosing the perfect remedial agent in reptiles is more complex than for mammals because of their variety of distinct characteristics: anatomical, physiological, and behavioral [13]. Information about antimicrobial efficiency in reptiles remains deficient, although studies on the efficiency of gentamicin [14], piperacillin [15], carbenicillin [16], ceftazidime [17], and enrofloxacin [18,19] are being identified in the literature, but are limited regarding reptilian cases and species.

Generally, the given dosages are either extrapolated from human medicine or empirically assumed according to the reptile species. A reason for this could be the lack of a proper anti-infective agent (or group) for the particular pathology of the reptiles, which may often cause an inaccurate use of another related class of antimicrobials for long periods [12,13]. With this aim, the phenomenon of resistance to antimicrobials commonly used in reptiles is gradually increasing, and the overall observed trend of this phenomenon is expanding. In reptiles, the common bacteria are considered commensal; infections usually include Gram-negative bacteria [20,21].

Knowledge about antibiotic resistance is of importance for disease prevention and control and from this point of view, reptile species illustrate relevant topics in exotic animal medicine since numerous entities found in reptiles have zoonotic reverberance [22]. The most frequently identified bacteria of pet reptiles are *Salmonella* spp., with reports on salmonellosis in reptiles being regularly identified in the literature [23,24,25,26]. Additionally, studies on *Clostridium*, *Mycobacterium*, *Campylobacter*, *Leptospira*, *Pseudomonas*, *Citrobacter*, *Klebsiella*, and *Proteus* spp. are found in the literature [27,28,29,30]. In reptilian species, the simplest and easiest way to combat resistance is the completion of an antibiogram, as a current and reliable method, to set up the best anti-infective remedy and to preserve the antimicrobial agents available for the therapy [12,13].

This examination emerged for practical reasons and the necessity for more information on this subject. The study was focused on the parallel analysis of pathologies responsible for the major diseases in various reptile species kept in terrariums as pets, with the aim of obtaining a better understanding of the main features of antibiotic therapy and antibiotic resistance in these species.

## 2. Materials and Methods

### 2.1. Sample Collection

The investigation took place over a period of three years in a specialized reptile clinic; 389 cases were presented for consultation, and treatment was performed during this time, namely, on 166 chelonians, 98 snakes, (venomous/non-venomous: 39/59), and 125 lizards. All reptiles with specific pathologies were regular patients of the clinic, and all owners were informed about this study which was, in general, harmful for the animals, participants consented in writing to participate.

The investigation objectives were to compare the pathology, therapy, and the bacterial load in the reptiles’ principal entities, namely, the skin and appendages; sensitive organs and the nervous system; the digestive system; the respiratory and cardiovascular system; the urinary system; genitalia; the osteo–muscular apparatus; surgical issues; tumors and intoxication, as presented in Supplementary Table S1.

Of the 389 reptiles presented for veterinary assistance that had different pathologies and were treated with antimicrobials, 25 individuals (2 chelonians, 8 lizards, and 15 snakes) did not show any clinical recovery following treatment. Therefore, a total of 43 samples were gathered for bacteriological examination, collected from the oral cavity, respiratory tract, skin wounds, abscesses, and feces, as presented in Table 1.

### 2.2. Bacterial Strain Identification

All bacterial strains isolated from reptiles were isolated using conventional methods, as specified in the protocols recommended by the samples, which were collected using the ESwab^TM^ (Copan, Brescia Italy) transport systems. Samples were then stored in cooling containers and transported, in accordance with the guidelines for biological sample collection and transport, to the research laboratory of transmissible diseases in pets (B.6.d); the samples were processed in the shortest possible time (max. 3 h post-collection).

The samples were processed in the Bacterial Diseases diagnostic laboratory (B.6.a), part of the Faculty of Veterinary Medicine’s Department of Infectious Diseases and Preventive Medicine in Timisoara. Collected samples moistened with sterile saline were inoculated onto BD Columbia Agar plates with 5% Sheep Blood (Becton Dickinson GmbH, Kelberg, Germany) and incubated at 37 °C for 24 h under aerobic conditions. The identification of bacterial strains in primary culture was based on colony morphology, appearance, type of hemolysis, and Gram staining. The specific colonies were inoculated on McConkey agar (Thermo Fisher Scientific, Loughborough, UK) using a bacteriological loop and incubated at 37 °C in an aerobic atmosphere for 24 h.

Following the current CLSI-30 standard [31] for the examination, the VITEK^®^2 automatic technique was used following the manufacturer’s instructions to identify the bacterial species. Gram-negative species were identified using the Vitek 2^®^ ID-GN card (bioMérieux. Marcy l’Etoile, France), designed for the automated identification of significant clinically fermenting and nonfermenting Gram-negative bacilli.

To identify Gram-positive bacteria, we inoculated VITEK 2^®^ ID-GP identification cards (bioMérieux, Marcy l’Etoile, France) according to the manufacturer’s instructions and analyzed and interpreted the results using the VT2-Software program, version R02. 03. The Vitek 2^®^ ID-GP card is a 64-well card designed for automated identification of most Gram-positive bacteria clinically significant in veterinary medicine.

The results were categorized as sensitive (S), intermediate (I), or resistant (R). 

Bacterial strains were gathered from reptiles with diverse pathogenies, using sterile cotton swabs moistened with sterile saline (Amies-Prima). The buffers were seeded on 5% blood agar (Biomedics SL, Madrid, Spain) and McConkey agar (Difco Laboratories, Franklin Lakes, NJ, USA) and then incubated at 37 °C, under aerobic conditions for 24 h. Following incubation, the colonies that grew on the 5% blood agar were identified morphologically and according to their tinctorial affinity. The catalase-positive coccoids and the Gram-positive strains were included in the *Staphylococcus* group and tested for their coagulase activity. In the same way, the Gram-negative bacteria were seeded on the XLD medium to obtain isolated colonies and pure cultures. The subcultures obtained were bacterioscopically controlled to verify the purity, and then from these colonies, seeding was performed on differential and selective media. To identify the clumping factor, a *Staphylococcus aureus* strain kit (Oxoid, Basingstoke Hampshire, UK) was used, with latex sensitized with fibrinogen and IgG contacting the fresh strains. Free coagulase was detected using rabbit and cattle citrated plasma and the Bactident Coagulase kit (Merck Millipore, Darmstadt, Germany).

### 2.3. Antimicrobial Susceptibility Testing Using VITEK^®^2 AST GN67 and GP69 Cards

Antimicrobial susceptibility testing of the isolated Gram-negative bacterial strains was achieved with the VITEK 2^®^ automated equipment and the AST GN67 card (bioMérieux. Marcy l’Etoile, France). The tested antimicrobials were: amikacin (AN; MIC range 16–64 μg/mL), ampicillin (AM; MIC range 8–32 μg/mL), ampicillin/sulbactam (SAM; MIC range 8/4–32/16 μg/mL), cefazolin (CZ; MIC range 2–8 μg/mL), cefepime (FEP; MIC range 2–16 μg/mL), ceftazidime (CAZ; MIC range 4–16 μg/mL), ceftriaxone (CRO; MIC range 1–4 μg/mL), ciprofloxacin (CIP; MIC range 0,06–1 μg/mL), ertapenem (ETP; MIC range 0,5–2 μg/mL), gentamicin (GM; MIC range 4–16 μg/mL), imipenem (IPM; MIC range 1–4 μg/mL), levofloxacin (LEV; MIC range 0.12–2 μg/mL), nitrofurantoin (FT; MIC range 32–128 μg/mL), piperacillin/tazobactam (TZP; MIC range 16/4–128/4 μg/mL), tobramycin (TM; MIC range 4–16 μg/mL) and trimethoprim/sulfamethoxazole (SXT; MIC range 2/32–4/76 μg/mL). The obtained results were automatically processed by the system, and the isolates were categorized as susceptible, resistant, or intermediate. The isolates resistant to three or more classes of antimicrobials were classified as multidrug-resistant.

The VITEK 2^®^, AST-GP69 Gram-positive specific bacteria card (bioMérieux, Marcy l’Etoile, France), was used to determine antibiotic sensitivities for Gram-positive bacteria strains isolated from reptiles with European Union (EU) drug configuration for companion animals. The study included a total of 19 antimicrobial substances (minimum inhibitory concentration [MIC]) from 13 different classes: ß lactams include benzylpenicillin (PCG; 0.03–0.5 g/mL), oxacillin (OXA; 0.25–4 g/mL), imipenem (IPM; 1–8 g/mL), ampicillin (AM; 2–64 g/mL), and ampicillin/sulbactam (SAM; 2–64 g/mL); aminoglycosides—gentamicin (GM; 0.5–16 µg/mL), kanamycin (K; 0.25–64 µg/mL); quinolones—enrofloxacin (ENR; 0.25–16 µg/mL), marbofloxacin (MBX; 0.25–8 µg/mL); steroids—fusidic acid (FUS; 1–16 µg/mL); vancomycin (VAN; 0.25–8 µg/mL); macrolides—erythromycin (ERY; 0.25–16 µg/mL µg/mL), rifamycins—rifampicin (RIF; 0.5–8 µg/mL); lincomycins—clindamycin (CLI; 0.25–16 µg/mL), tetracyclines—tetracycline (TE; 2–32 µg/mL); sulfonamides—trimethoprim/sulfamethoxazole (SXT; 20–76 µg/mL); nitrofuran derivate—nitrofurantoin (FT; 16–512 µg/mL); pseudomonic acid derivatives—mupirocin (MUP; 0.06–512 µg/mL) and amphenicols—chloramphenicol (CHL; 4–32 µg/mL). The MIC at which a bacterial isolate is considered susceptible is according to CLSI guidelines, CLSI M31-A4 2013.

Following the CLSI specified guidelines, quality control was performed using *Staphylococcus aureus* ATCC^®^23235™ and *Pseudomonas aeruginosa* ATCC 27853™. Antimicrobial susceptibility results obtained from quality control strains were within the ranges established for quality control.

### 2.4. Interpretation of Results

The results were categorized in confidence levels, with the identification percentage ranging from 99.9 to 80.0%, as: excellent, very good, good to satisfactory, and matching a distinct profile, compared with others from the database. Next, the obtained value was extrapolated to the t-index, an algorithm that estimates the profile’s closeness to the most typical response to each bacterial profile. The t-index can vary between 0 and 1 and is inversely proportional to the number of atypical tests. Therefore, an excellent trust level is an identifier of 99.9% and a t-index of 0.75 combinations; an acceptable confidence level combines an identification rate of 80.0% and a t-index of 0; in the case of low-level differentiation, additional tests are proposed. All characterized isolates in our study have shown very good (%ID ≥ 99.0, T index ≥ 0.5) confidence levels.

### 2.5. The Statistical Analysis

Graph Pad Prism 9.0 for Windows (Graph Pad software, San Diego, CA, USA) was utilized as the statistical software. The mean SEM (standard error of the mean) was used to express all data. For accuracy, two-way analysis of variance (ANOVA) with Tukey’s multiple comparison tests and Bonferroni correction were used to determine the difference and statistical significance between groups. Differences were considered significant as follows: * means 0.01 ≤ *p* < 0.05 significant; ** means 0.001 ≤ *p* < 0.01 highly significant and *** means *p* < 0.001 very highly significant; ns: indicates not significant.

## 3. Results

### 3.1. Main Pathology Analysis of the Reptiles

Of the 389 reptiles studied, we identified nine pathological entities. Five of these were major: pathologies of the digestive system, skin, the respiratory system, CNS/sensory organs, and the reproductive system; there were four additional pathological entities, but in a much smaller proportion: pathologies of the osteo–muscular system (of a medical/traumatic nature), the urinary system, tumors, and intoxication.

**Figure 1:**
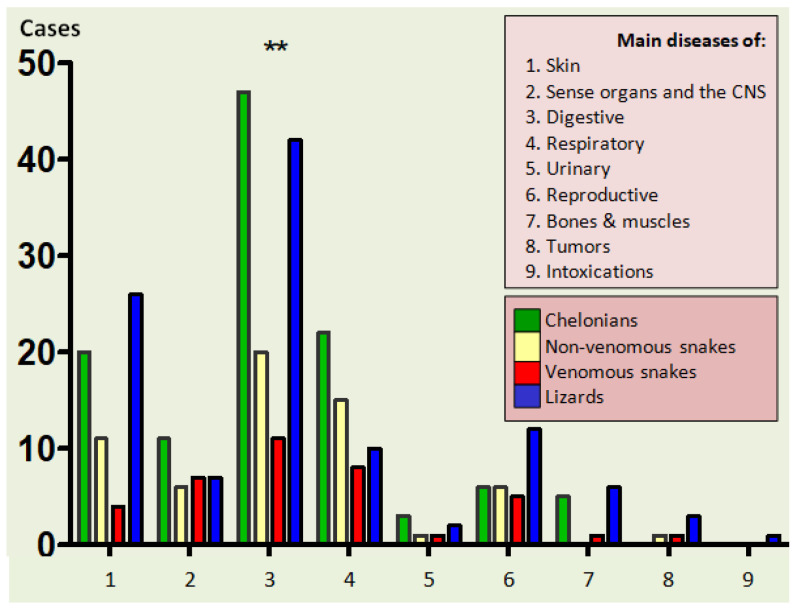
Comparing the incidence of digestive diseases and other diseases within the reptile category as diagnosed in this study (where: ** means *p* < 0.01).

A statistically significant relationship linking disease incidence in the reptile species was discovered and expressed in all of the species and diseases in this experiment. The digestive dysfunction was most frequently recognized (*p* < 0.01). The relationship between digestive disease incidence and that of the other diseases was confirmed to be highly significant (*p* < 0.001) (Figure 1).

**Figure 2:**
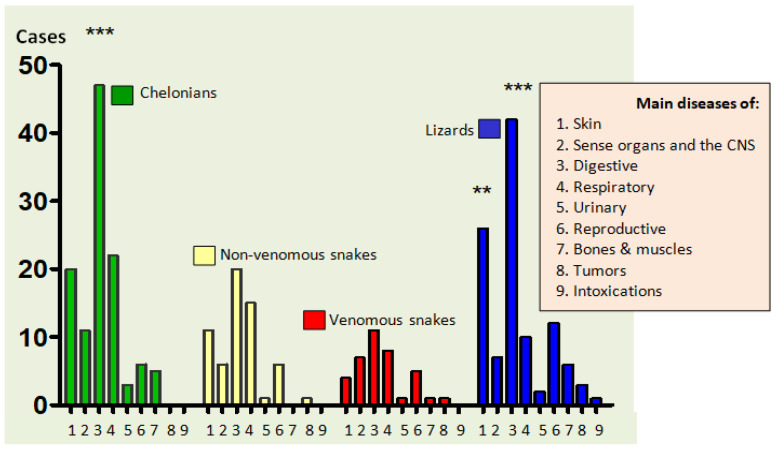
Comparative disease distribution/no. of cases/reptile species/disease types; comparison of chelonians vs. venomous and non-venomous snakes (where *** means *p <* 0.001) and lizards vs. venomous and non-venomous snakes *(*where ** means *p* < 0.01, and *** means *p <* 0.001).

The comparative distribution of diseases/number of cases/reptile species/type of disease revealed statistically significant and highly significant values for the following: chelonians vs. venomous and non-venous snakes (where ***, means *p <* 0.001) and lizards vs. venomous and non-venomous snakes (where **, means *p* < 0.01 and *** means *p* < 0.001) (Figure 2).

**Figure 3:**
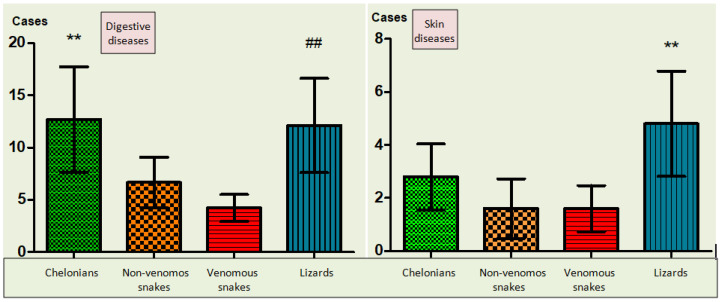
The incidence of digestive diseases (the most common—left image) and skin diseases (the second most common—right image); comparison of chelonians vs. snakes (where ** means *p* <0.01); lizards/snakes *(*where ## means *p <* 0.01) and lizards vs. chelonians and snakes *(*where ** means *p <* 0.01.).

By examining the relationship and associating the reptile species according to the two main diseases identified (digestive and dermatological), it was observed that the frequencies of digestive (most observed) and skin (the second most commonly disease) diseases were increased in chelonians vs. snakes (*p* < 0.01); lizards vs. snakes (*p* < 0.01) and lizards vs. chelonians and snakes (*p* < 0.01) (Figure 3).

The comparative correlations made between reptile species/disease type for chelonians vs. snakes vs. lizards were revealed to be highly statistically significant (p < 0.001), which, in this case, indicates that the most sensitive reptile species kept in terrariums can be organized in order: chelonians, venomous snakes, non-venomous snakes, and lizards.

### 3.2. Frequency of Bacterial Resistance to Antibiotics

The most commonly isolated bacterial strains were Enterococcus faecalis (six cases), Pseudomonas aeruginosa (five cases), Stenotrophomonas (Xanthomonas) maltophilia (four cases), E. coli (three cases), Klebsiella oxytoca spp. (two cases), Beta-hemolytic streptococci, Staphylococcus aureus, Citrobacter spp., and Proteus spp. (one case).

The bacteria isolated from the reptiles exhibited diverse degrees of resistance against most of the antimicrobials, including cephalosporins (cefalexin, cefuroxime, and cefquinome), macrolides (erythromycin), lincosamides, penicillins, (ampicillin, amoxicillin/clavulanic acid, and amikacin), florfenicol, tetracyclines (tetracycline and doxycycline), and aminoglycosides (gentamycin). Resistance was less commonly reported for chloramphenicol, sulfonamides, and quinolones (Table 2).

According to the frequency, resistance manifested as follows:

- Most common in *Pseudomonas aeruginosa*, followed by *Citrobacter brakii*, *Enterococcus faecalis,* and *Stenotrophomas (Xanthomonas) maltophilia*.
- Relatively common in *Citrobacter freundi*, *Acinetobacter lwoffii,* and *Salmonella* spp.
- Less common in *Clostridium fallax*, *Staphylococcus aureus* (resistance was absent), *Proteus mirabilis*, *Delftia acidovorans,* and *Morganella morganii* spp., *morganii*.

## 4. Discussion

The importance of this study stems from the fact that some isolated bacterial pathogens represent an important zoonotic risk and are considered a potential reservoir for resistant bacteria in the human owners of these pets, confirming other research from the mainstream scientific literature [2,9,11,12,29].

In the last decade, there has been an increasing trend in the European Union (EU) in that, currently, the EU region is the largest importer of reptiles globally [32], spawning studies on topics discussing the recrudescence of new and diverse forms of infection, especially those of zoonotic, bacterial [33,34], viral [35], parasitological [36], or fungal origin [37] in pet reptiles.

The emergence of these infections fully justifies the use of antimicrobials, but long-term antimicrobial treatments have undoubtedly influenced the evolution of resistance; however, this has unfortunately not yet been thoroughly identified in pet reptile species [38], thus justifying the present study, which, to the best of our knowledge, is the first of its kind in Romania.

This is why anti-infectious treatment often fails, particularly in cases when antibiotics are used without antibiotic sensitivity confirmation [11,21,22]. Therefore, resistance to routinely used antibiotics in reptiles is increasing and can be considered to be frequent, confirming our obtained results.

In this research, almost all of the antimicrobials tested exhibited increases in resistance. In the quinolone group, two strains were identified in two cases; in the macrolide group, 19 strains out of 22 were identified.

The identified bacteria displayed resistance against the majority of the commonly used antibiotic combinations, including those used in this study: penicillins (ampicillin and amoxicillin/clavulanic acid), cephalosporins (cefalexin, cefuroxime, and cefquinome), macrolides (erythromycin), lincosamides, amikacin, gentamycin, and tetracyclines (tetracycline and, doxycycline). Other authors reported similar results to those obtained in this study [21,22].

The comparative distribution of diseases/number of cases/reptile species/types of disease revealed statistically significant and highly significant values for chelonians vs. venomous and non-venomous snakes (*p* < 0.001) and lizards/venomous/non-venomous snakes (*p* < 0.01), respectively (*p*< 0.001).

In this study, we observed significant statistical correlations (*p* < 0.01) between disease incidence and reptile species, with digestive diseases being the most frequent. The obtained results agree, to a large extent, with those presented by van Zanten and Simpson and Lee in their reviews [38,39].

## 5. Conclusions

The most common pathological entity found in reptiles was digestive (medical/parasitic) pathology. The other main pathological entities found were related to (in the following order): the skin, the sensory organs, the digestive system, the respiratory system, the cardiovascular system, the urinary system, the genitalia, the osteo–muscular tract, surgical issues, tumors, and intoxications.

The animals can be categorized according to their sensitivity to diseases in the following order (most sensitive to least sensitive): chelonians, venomous snakes, non-venomous snakes, and lizards.

## Figures and Tables

**Table 1:** Presentation of cases: bacterial media, reptiles, and antibiotics tested.

No.	Bacterial Strain Identified	Isolates	From Total%	Sampled From	Reptile Species
1	*Achromobacter spanius*	1	2.32	oral cavity	*Trachemys scripta scripta*
2	*Acinetobacter lwoffii*	1	2.32	respiratory tract	*Testudo hermanni*
3	*Bacillus pumilus*	1	2.32	s.c. abscess	*Iguana iguana*
4	*Citrobacter amalonaticus*	1	2.32	oral cavity	*Varanus cumingi*
5	*Citrobacter brakii*	1	2.32	oral cavity	*Python regius*
6	*Citrobacter freundii*	2	4.65	trachea	*Morelia spilota*
				feces	*Cordylus cataphractus*
7	*Citrobacter koseri*	1	2.32	dermal wound	*Iguana iguana*
8	*Clostridium fallax*	1	2.32	trachea	*Boa constrictor*
9	*Delftia acidovorans*	1	2.32	oral cavity	*Python regius*
10	*Enterobacter cloacae*	1	2.32	trachea	*Pogona vitticeps*
11	*Enterococcus casseliflavus*	2	4.65	respiratory tract	*Testudo hermanni*
				oral cavity	*Bitis arietans*
12	*Enterococcus faecalis*	6	13.95%	oral cavity	*Pseudocerastes persicus*
				oral cavity	*Python regius*
				trachea	*Vipera latastei*
				trachea	*Vipera orlovi*
				s.c. abscess	*Iguana iguana*
				dermal abscess	*Pogona vitticeps*
13	*Escherichia coli*	3	6.97	oral cavity	*Morelia spilota*
				trachea	*Varanus cumingi*
				trachea	*Pseudocerastes persicus*
14	*Klebsiella oxytoca*	3	6.97	trachea	*Morelia spilota*
				oral cavity	*Pogona vitticeps*
				dermal abscess	*Pogona vitticeps*
15	*Morganella morganii* spp. *morganii*	1	2.32	trachea	*Morelia spilota*
16	*Proteus mirabilis*	2	4.65	dermal abscess	*Pogona vitticeps*
				feces	*Cordylus cataphractus*
17	*Proteus vulgaris*	2	4.65	trachea	*Trachemys scripta scripta*
				kidney	*Bitis schneideri*
18	*Pseudomonas aeruginosa*	5	11.62	trachea	*Morelia spilota*
				oral cavity	*Vipera orlovi*
				oral cavity	*Python regius*
				dermal abscess	*Pogona vitticeps*
				dermal wound	*Iguana iguana*
19	*Salmonella* spp.	2	4.65	oral cavity	*Bitis schneideri*
				feces	*Cordylus cataphractus*
20	*Staphylococcus aureus*	1	2.32	oral cavity	*Pogona vitticeps*
21	*Stenotrophomas maltophilia*	4	9.30	trachea	*Pantherophis guttatus*
				trachea	*Python regius*
				oral cavity	*Vipera latastei*
				oral cavity	*Python regius*
22	*Streptococi beta haemolitici*	1	2.32	s.c. formation at the tail’s base	*Pogona vitticeps*

Antimicrobial susceptibility testing using VITEK^®^2 AST GN67 and GP69 cards. Beta-lactams and Cephalosporins: Amoxicillin; Amikacin; Penicillin; Oxacillin; Cefazolin; Cefaclor; Cefalexin; Cephalothin; Cefuroxime; Ceftazidime; Cefovecin; Cefquinome; Aminoglycosides: Gentamicin; Kanamycin; Neomycin; Macrolides: Erythromycin, Azithromycin; Tulathromycin; Lincomycin; Clindamycin; Mupirocin; Fucidin; Quinolones: Enrofloxacin; Ciprofloxacin; Marbofloxacin; Orbifloxacin; Pradofloxacin; Tetracyclines and Chloramphenicol: Tetracycline; Doxycycline; Chloramphenicol; Florphenicol; Polymyxins: Polymyxin B, Colistin; Sulfonamides and Trimethoprim.

**Table 2:** Results of antibiogram/distribution of bacterial strains (susceptible/resistant/antibacterial) used.

Isolated Bacterial Strains/No.				Strains	Antibiotic/Group/Generation												
1. *Pseudomonas aeruginosa*				**5**	**PEN**	**CEF1**	**CEF2**	**CEF3**	**CEF4**	**MAC**	**LINC**	**TETR**	**S + T**	**CLO**	**FLO**	**AMGL**	**QUIN**
2. *Citrobacter koseri*				**1**	**PEN**	**CEF1**	**CEF2**	**CEF3**	**CEF4**	**MAC**	**LINC**	**TETR**	**CLO**	**FLO**	**AMGL**	**QUIN**	**S + T**
*3. Citrobacter brakii*				**1**	**PEN**	**S + T**	**CLO**	**CEF3**	**CEF4**	**MAC**	**LINC**	**FLO**	**QUIN**	**AMGL**	**TETR**	**CEF1**	**CEF2**
4. *Enterococcus casseliflavus*				**2**	**CEF1**	**CEF2**	**CEF3**	**CEF4**	**MAC**	**LINC**	**AMGL**	**TETR**	**S + T**	**PEN**	**QUIN**	**CLO**	**FLO**
5. *Enterococcus faecalis*				**6**	**CEF1**	**CEF2**	**CEF3**	**CEF4**	**MAC**	**LINC**	**AMGL**	**TETR**	**S + T**	**PEN**	**QUIN**	**CLO**	**FLO**
6. *Klebsiella oxytoca*				**3**	**PEN**	**CEF1**	**CEF2**	**CEF3**	**CEF4**	**MAC**	**LINC**	**TETR**	**FLO**	**S + T**	**CLO**	**AMGL**	**QUIN**
7. *Stenotrophomas(Xanthomonas)maltophilia*				**4**	**PEN**	**CEF1**	**CEF2**	**CEF3**	**CEF4**	**MAC**	**LINC**	**AMGL**	**FLO**	**QUIN**	**TETR**	**S + T**	**CLO**
8. *Proteus vulgaris*				**2**	**PEN**	**CEF1**	**CEF2**	**CLO**	**TETR**	**MAC**	**LINC**	**AMGL**	**QUIN**	**S + T**	**CEF3**	**CEF4**	**FLO**
9. *Citrobacter freundii*				**2**	**PEN**	**CEF1**	**TETR**	**FLO**	**MAC**	**LINC**	**AMGL**	**QUIN**	**S + T**	**CLO**	**CEF2**	**CEF3**	**CEF4**
10. *Enterobacter cloacae*				**1**	**PEN**	**CEF1**	**CEF2**	**MAC**	**LINC**	**TETR**	**AMGL**	**QUIN**	**CEF3**	**CEF4**	**S + T**	**CLO**	**FLO**
11. *Acinetobacter lwoffii*				**1**	**PEN**	**CEF1**	**CEF2**	**MAC**	**LINC**	**FLO**	**AMGL**	**QUIN**	**TETR**	**S + T**	**CLO**	**CEF3**	**CEF4**
12. *Achromobacter spanius*				**1**	**PEN**	**S + T**	**CLO**	**FLO**	**MAC**	**LINC**	**AMGL**	**QUIN**	**TETR**	**CEF1**	**CEF2**	**CEF3**	**CEF4**
13*. Salmonella* spp.				**2**	**FLO**	**CEF1**	**CEF2**	**MAC**	**LINC**	**AMGL**	**QUIN**	**TETR**	**S + T**	**CLO**	**CEF3**	**CEF4**	**PEN**
14. *Escherichia coli*				**3**	**PEN**	**CEF1**	**FLO**	**MAC**	**LINC**	**CEF2**	**QUIN**	**TETR**	**S + T**	**CLO**	**CEF3**	**CEF4**	**AMGL**
15. *Morganella morganii spp. morganii*				**1**	**PEN**	**MAC**	**LINC**	**FLO**	**TETR**	**CEF3**	**CEF4**	**AMGL**	**QUIN**	**S + T**	**CLO**	**CEF1**	**CEF2**
16. *Citrobacter amalonaticus*				**1**	**PEN**	**MAC**	**LINC**	**TETR**	**FLO**	**CEF3**	**CEF4**	**AMGL**	**QUIN**	**S + T**	**CLO**	**CEF1**	**CEF2**
17. *Delftia acidovorans*				**1**	**PEN**	**MAC**	**LINC**	**QUIN**	**TETR**	**S + T**	**CLO**	**AMGL**	**FLO**	**CEF1**	**CEF2**	**CEF3**	**CEF4**
18. *Streptococi beta hemolitici*				1	**MAC**	**LINC**	**AMGL**	**QUIN**	**TETR**	**S + T**	**CLO**	**FLO**	**PEN**	**CEF4**	**CEF1**	**CEF2**	**CEF3**
19*. Proteus mirabilis*				2	**MAC**	**LINC**	**TETR**	**PEN**	**CEF1**	**CEF2**	**CEF3**	**CEF4**	**AMGL**	**QUIN**	**S + T**	**CLO**	**FLO**
20*. Bacillus pumilus*				1	**TETR**	**MAC**	**LINC**	**PEN**	**CEF1**	**S + T**	**CLO**	**AMGL**	**QUIN**	**CEF2**	**CEF3**	**CEF4**	**FLO**
21. *Staphylococcus aureus*				1	**PEN**	**CEF1**	**TETR**	**S + T**	**CLO**	**MAC**	**LINC**	**AMGL**	**QUIN**	**CEF2**	**CEF3**	**CEF4**	**FLO**
22. *Clostridium fallax*				1	**PEN**	**MAC**	**TETR**	**CLO**	**LINC**	**AMGL**	**QUIN**	**S + T**	**FLO**	**CEF1**	**CEF2**	**CEF3**	**CEF4**
**R**	**S**	**I**	Total	**43** Isolated bacterial strains													

**Legend:** R—resistant; S—sensitive; I—intermediate. PEN = penicillin; CEF 1, 2, 3, 4 = cephalosporin (from generation 1, 2, 3 or 4); MAC = macrolides; LINC = lincosamides; AMGL = aminoglycosides; QUIN = quinolones; TETR = tetracycline; S + T = sulfonamides + trimethoprim; CLO = chloramphenicol; FLO = florphenicols.

## Data Availability

Not applicable.

## Supplementary Materials

The following supporting information can be downloaded at: https://www.mdpi.com/article/10.3390/ani12101279/s1, Table S1: The specific pathology found/categories treated/reptile species.
