# Supplementary material for: Pathology and Prevalence of Antibiotic-Resistant Bacteria: A Study of 398 Pet Reptiles
Source: Animals (Basel). 2022 May 17;12(10):1279. doi: 10.3390/ani12101279 (PMC9137941; doi:10.3390/ani12101279)
Supplement: Supplementary file 1 [file animals-12-01279-s001.zip › animals-1695442-supplementary.pdf]

Table S1. *The specific pathology found / categories treated / reptile species*

Categories of treated pathologies / reptile species	Chelonians	Snakes *Venomous*	Snakes *Non-venomous*	Lizards
Skin & appendages	20	4	11	26
Sensory organs and Nervous system	11	7	6	7
Digestive system	99	11	20	58
Respiratory & cardio / vascular apparatus	22	8	15	10
Urinary system	3	1	1	2
The breeding apparatus	6	5	6	12
Osteo-muscular apparatus	5	1	0	6
Tumors	-	1	1	3
Intoxications	-	-	-	1

Of which by species of reptile:

**Chelonians**	N	Snakes: *Venomous*	N	Snakes: *Non-venomous*	N	**Lizards**	N
Testudo hermanni	13	*Bitis arietans*	1	*Python regius*	1	*Pogona vitticeps*	7
Testudo graeca	9	*Bitis nasicornis*	5	*Pantherophis guttatus*	1	*Iguana iguana*	1
Testudo horsfieldii	5	*Vipera transcaucasiana*	3	*Boa constrictor*	1	*Eublepharis macularius*	7
Testudo marginata	4	*Agkistrodon bilineatus*	2	*Morelia spilota*	5	*Tupinambis teguixin*	4
Trachemys scripta elegans	4	*Pseudocerastes persicus*	3	*Aspidites ramsay*	3	*Gekko grossmanni*	3
Pseudemys spp.	2	*Crotalus polystictus*	2	*Coronella austriaca*	2	*Uromastyx acanthinura*	3
Chelodina longiccolis	1	*Cerastes cerastes*	2	*Lampropeltis californiae*	2	*Crotaphytus collaris*	2
Graphtemys pseudogeografica	1	*Vipera orlovi*	2	*Morelia viridis*	2	*Furcifer pardalis*	2
Stigmochelys pardalis	1	*Vipera kaznakovi*	2	*Boa constrictor imperator*	1	*Gambelia wislizenii*	2
Terrapene ornata	1	*Bitis gabonica*	1	*Corallus caninus:*	1	*Varanus cumingi*	2
Centrochelys sulcata	1	*Bitis schneideri*	1	*Heterodon nasicus*	1	*Heloderma suspectum*	2
		Crotalus lepidus morulus	1	*Python morulus bivittatus*	1	*Acanthosaura lepidogaster*	1
		Gloydius halys	1	*Python morulus morulus*	1	*Chamaeleo calyptratus*	1
		Malpolon monspessulanus	1	*Zamenis situla*	1	*Cordylus catophractus*	1
		Montivipera xanthina	1			*Ctenosaura similis*	1
		Protobotrops cornuta	1			*Tiliqua scincoides*	1
		Vipera aspis	1				
**Total turtles: 166**		**Venomous: 39**		**Non-venomous: 59**			
		**Total snakes: 98**				**Total lizards: 125**	

**Total reptiles: 389**
